# Mispositioned Neurokinin-1 Receptor-Expressing Neurons Underlie Heat Hyperalgesia in *Disabled-1* Mutant Mice

**DOI:** 10.1523/ENEURO.0131-19.2019

**Published:** 2019-06-19

**Authors:** Xidao Wang, Griselda M. Yvone, Marianne Cilluffo, Ashley S. Kim, Allan I. Basbaum, Patricia E. Phelps

**Affiliations:** 1 Departments of Anatomy and Physiology and W. M. Keck Foundation Center for Integrative Neuroscience, University of California, San Francisco, CA 94158; 2 Department of Integrative Biology and Physiology UCLA, Los Angeles, Los Angeles, CA 90095

**Keywords:** Dab1, lateral spinal nucleus, Lmx1b, pain, *reeler*, superficial dorsal horn

## Abstract

Reelin (Reln) and Disabled-1 (Dab1) participate in the Reln-signaling pathway and when either is deleted, mutant mice have the same spinally mediated behavioral abnormalities, increased sensitivity to noxious heat and a profound loss in mechanical sensitivity. Both Reln and Dab1 are highly expressed in dorsal horn areas that receive and convey nociceptive information, Laminae I–II, lateral Lamina V, and the lateral spinal nucleus (LSN). Lamina I contains both projection neurons and interneurons that express Neurokinin-1 receptors (NK1Rs) and they transmit information about noxious heat both within the dorsal horn and to the brain. Here, we ask whether the increased heat nociception in *Reln* and *dab1* mutants is due to incorrectly positioned dorsal horn neurons that express NK1Rs. We found more NK1R-expressing neurons in *Reln^-/-^* and *dab1^-/-^* Laminae I–II than in their respective wild-type mice, and some NK1R neurons co-expressed Dab1 and the transcription factor Lmx1b, confirming their excitatory phenotype. Importantly, heat stimulation in *dab1^-/-^*mice induced Fos in incorrectly positioned NK1R neurons in Laminae I–II. Next, we asked whether these ectopically placed and noxious-heat responsive NK1R neurons participated in pain behavior. Ablation of the superficial NK1Rs with an intrathecal injection of a substance P analog conjugated to the toxin saporin (SSP-SAP) eliminated the thermal hypersensitivity of *dab1^-/-^* mice, without altering their mechanical insensitivity. These results suggest that ectopically positioned NK1R-expressing neurons underlie the heat hyperalgesia of Reelin-signaling pathway mutants, but do not contribute to their profound mechanical insensitivity.

## Visual Abstract

**Figure:**
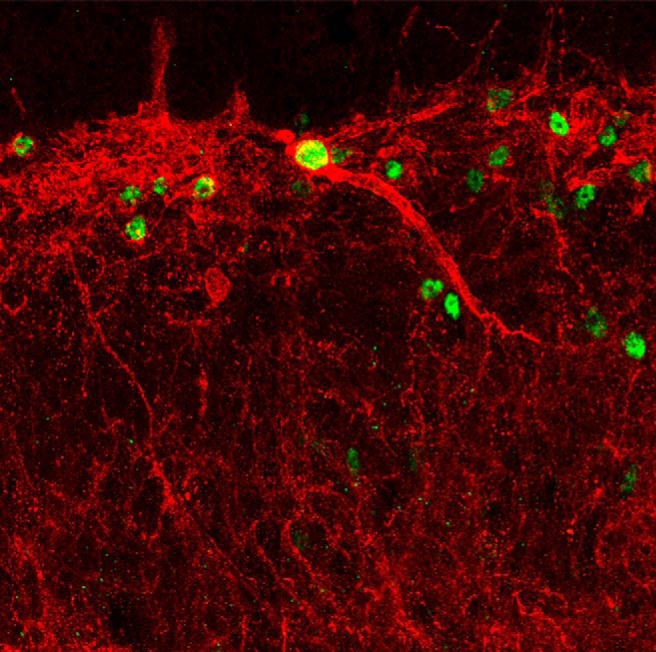

## Significance Statement

Mutants deficient in Reelin signaling have increased sensitivity to noxious heat and decreased mechanical sensitivity, but the underlying mechanisms are unknown. Here, we ask whether the heat hyperalgesia results from additional activation of misplaced dorsal horn neurons that express Neurokinin-1 receptors (NK1Rs). Compared to wild-type mice, *dab1^-/-^* mice have more NK1R-expressing neurons in the superficial dorsal horn and these neurons express Fos in response to noxious heat stimulation. Ablation of these neurons by intrathecal injection of a substance P analog conjugated to saporin (SSP-SAP) abolished the hypersensitivity of the heat responses of mutant mice, but the mechanical insensitivity was preserved. Mispositioned NK1R-expressing neurons, therefore, underlie the heat hyperalgesia that characterizes Reelin-signaling mutant mice.

## Introduction

Reelin (Reln) is a secreted glycoprotein that binds both the apolipoprotein E receptor 2 (Apoer2) and the very-low-density lipoprotein (Vldl) receptors. Reln binding, in turn, leads to receptor clustering, followed by recruitment and phosphorylation of the intracellular adaptor protein Disabled-1 (Dab1) by Src-family kinases (D'Arcangelo et al., 1999; Herz and Chen, 2006). Mice with mutations in *Reln*, or both *Apoer2* and *Vldlr*, or *Dab1* have similar patterns of mispositioned neurons generated during nervous system development (Rice and Curran, 1999; Trommsdorff et al., 1999). The Reln-Dab1 signaling pathway regulates neuronal positioning in the spinal cord in a cell-specific manner. Neurons that express Dab1 sustain migratory errors in mutant mice, whereas other neurons are usually appropriately positioned (Phelps et al., 2002).

Reln-expressing and Dab1-expressing neurons are concentrated in the superficial dorsal horn (Laminae I–II) and in the lateral reticulated area of Lamina V, where nociceptive afferents terminate and contain projection neurons that integrate and convey nociceptive signals to supraspinal targets (Villeda et al., 2006; Akopians et al., 2008; Basbaum et al., 2009; Todd, 2010). The lateral spinal nucleus (LSN), a small area embedded in the dorsolateral funiculus that transmits noxious information to the brainstem and thalamus (Burstein et al., 1990; Olave and Maxwell, 2004), also contains Reln-labeled and Dab1-labeled neurons (Villeda et al., 2006). Importantly, functional abnormalities characterize *Reln^-/-^* and *dab1^-/-^* mice: an increased sensitivity to noxious heat (hyperalgesia) and a pronounced reduction in mechanical and chemical sensitivity. Cold and visceral pain transmission, however, appeared normal (Villeda et al., 2006; Akopians et al., 2008; Wang et al., 2012). Taken together, these findings indicate that the biological basis of different pain modalities is segregated in mutants of the Reln-signaling pathway.

Despite these profound nociceptive abnormalities in the processing of pain messages, the alterations in the dorsal horn that underlie these opposite sensitivities to heat and mechanical stimuli are unclear. Although the termination pattern of nociceptive primary afferents is generally similar in wild-type and mutant mice, *Reln^-/-^* Lamina I is compacted and the area of Laminae I–II outer is smaller than in *Reln^+/+^* mice (Villeda et al., 2006; Yvone et al., 2017). In addition, the isolectin B4 (IB4) terminal field that defines Lamina II dorsal inner appears shifted dorsally in *Reln^-/-^* yet contains greater numbers of Dab1-expressing neurons than found in *Reln^+/+^* mice (Yvone et al., 2017).

More relevant to the heat hypersensitivity is our report of misplaced Dab1 cells that express Neurokinin-1 receptors (NK1Rs) in *Reln^-/-^*and *Apoer2^-/-^/Vdlr^-/-^* mice (Villeda et al., 2006; Akopians et al., 2008). The NK1R-expressing neurons are targeted by the peptidergic (substance P-expressing) primary afferents (C-fibers; Al Ghamdi et al., 2009), respond to noxious heat stimulation, and the great majority are projection neurons that transmit noxious thermal information rostrally (Littlewood et al., 1995; Marshall et al., 1996; Spike et al., 2003). After ablation of NK1R-expressing neurons with the cytotoxic substance P-saporin conjugate (SP-SAP), responses to acute pain were unchanged but the thermal hyperalgesia associated with chronic pain was reduced (Mantyh et al., 1997; Nichols et al., 1999). Here, we asked whether the noxious heat hypersensitivity detected in *Reln^-/-^* and *dab1^-/-^* mice is related to the mispositioned neurons that express NK1Rs.

We first examined the laminar distribution of NK1R-expressing neurons in wild-type and mutant *Reln* and *dab1* dorsal horns and then asked whether the NK1R-expressing neurons were part of the Dab1 and Lmx1b populations. Next, we asked whether there were fewer NK1R-positive cells that expressed the immediate early gene, c-*fos,* after noxious heat stimulation in *dab1^+/+^*compared to *dab1^-/-^*Laminae I–II and LSN. Finally, to determine whether mispositioned NK1R-expressing neurons are responsible for the heat hypersensitivity in *dab1^-/-^*mice, we selectively ablated the superficially located NK1R-expressing neurons. We report that the ablation of NK1R-expressing neurons abolished the thermal hypersensitivity in *dab1^-/-^*mice, without altering their mechanical deficits.

## Materials and Methods

### Animals

All experiments were reviewed and approved by the Chancellor’s Animal Research Committee at University of California, Los Angeles (UCLA) or the Institutional Care and Animal Use Committee at the University of California, San Francisco and conducted in accordance with the National Institutes of Health Guide for the Care and Use of Laboratory animals. Adult *reeler* (B6C3Fe -*ala- Reln^rl^*, The Jackson Laboratory) and *dab1* KO mice (BALB⁄cByJ *dab1* ^–/–^, gifts from Dr. J. Cooper, Fred Hutchinson Cancer Research Center, Seattle, WN, and Dr. B. Howell, SUNY Upstate Medical University, Syracuse, NY; Howell et al., 1997) were bred at UCLA and genotyped using polymerase chain reaction as described by D’Arcangelo et al. (1996) and Brich et al. (2003). For anatomic analyses, adult mice (4–12 months) of either sex were deeply anesthetized with Pentobarbital (80 mg/kg), perfused with 4% paraformaldehyde, and postfixed for 3–4 h in the same fixative at 4°C. Lower lumbar spinal cord segments (L4-5) were dissected for all experiments and cervical enlargements (C5-8) were collected for studies in which we ablated NK1R-expressing neurons. Spinal cords were infiltrated with 30% sucrose, embedded and stored at –80°C.

### Behavioral assays

#### Thermal (heat)-induced Fos expression

We used six *dab1^+/+^* and six *dab1^-/-^* age-matched male mice to compare the positions of the NK1R-expressing neurons activated by noxious heat. As the *dab1^-/-^* mice are larger, healthier, and had higher Fos expression than the *Reln^-/-^* mice, we used *dab1* mice for testing (Wang et al., 2012). Mice were lightly anesthetized with pentobarbital (50–60 mg/kg) and 15 min later the left hindpaw was dipped into a 50°C water bath for 3 s/min for 10 min. One hour later, the mice were anesthetized (80–100 mg/kg) and perfused.

#### Hargreaves test of thermal (heat) sensitivity

Male *dab1* mice, seven of each genotype, were tested with the Hargreaves’ paw withdrawal test of heat sensitivity (Cao et al., 1998). Mice were placed in plastic chambers on a warm glass surface for 1 h, after which we focused a radiant heat source on the left hindpaw, and recorded the withdrawal latency.

#### von Frey test of mechanical sensitivity

Male *dab1* mice (seven of each genotype) were placed in clear plastic chambers on an elevated wire platform and habituated for 1 h. We used the up-down method (Chaplan et al., 1994) with calibrated monofilaments (0.008–4 g) to determine the mechanical withdrawal threshold. After the 0.4 filament was applied to the center of the left hindpaw, responses were recorded in series, and the 50% withdrawal threshold was determined.

#### Substance P analog conjugated saporin (SSP-SAP)

To determine whether mispositioned neurons that express NK1Rs in Laminae I–II and the LSN contribute to the heat hypersensitivity of *dab1^-/-^* mice, we selectively ablated these neurons by intrathecal injections of a peptidase-resistant analog of substance P [Sar^9^, Met (O_2_)^11^, SSP] conjugated to the cytotoxic agent saporin (SSP-SAP; Kit-11-25, Advanced Targeting Systems). Initially we tested wild-type mice with a range of concentrations (100–400 ng in 1-µl sterile PBS) for lumbar level intrathecal injections of SSP-SAP or control, unconjugated SAP. We found that 300 ng of SSP-SAP effectively killed NK1R-expressing neurons in lumbar superficial dorsal horn; the same concentration of unconjugated SAP had no effect.

Next, we examined thermal and mechanical responses using the Hargreaves and von Frey tests, respectively, both before (baseline) and after the ablations. We injected seven male *dab1^+/+^* and seven *dab1^-/-^* mice intrathecally with 300 ng of SSP-SAP. One month after SSP-SAP treatment we repeated the tests of heat and mechanical sensitivity with seven *dab1^+/+^* and six *dab1^-/-^* mice. Mice were then perfused and their spinal cords immunostained for NK1Rs.

#### Hot plate test of thermal sensitivity

We also tested the mice in a model of heat pain sensitivity that is presumed to involve circuits in the brain and spinal cord. Unfortunately, because of profound motor instability or “reeling” of the mutants, these tests proved very difficult. By videotaping the mice and observing different behavioral responses on the hot plate, however, we were able to identify behavioral endpoints that distinguished wild-type and mutant mice. We conducted the hot plate test (1440 Analgesia Hot Plate, Columbus Instruments) using 22 *dab1* female mice, evenly divided between genotypes. After a 30-min habituation period, mice received three trials each at 33°C baseline, 48°C, 52.5°C, and 55°C. Time to the first positive response was recorded, based on standard responses (shaking, licking, shielding hindpaw, or jumping), and alternate responses observed primarily, but not exclusively, in mutants, such as licking the trunk or leg, or intense grooming (anxious shaking or grooming that lasted ≥3 s or occurred three times). Unfortunately, we were not able to assess these mice after ablation of dorsal horn NK1R-expressing neurons, so we only report results from the untreated mice.

### Tissue preparation and immunohistochemical procedures

The number of cells with NK1Rs in L4-5 spinal cord was analyzed in transverse 40 µm cryostat sections. Free-floating sections were washed in PBST buffer (0.5 M PBS + 0.3% Triton X-100), and blocked for 1 h in 3% normal goat serum in PBST. Sections were incubated in anti-rabbit NK1R (1:10,000; Sigma-Aldrich catalog #S8305, RRID:AB_261562) overnight, rinsed in PBST, and placed in biotinylated anti-rabbit IgG (1:200; Vector Laboratories catalog #PK-6101, RRID:AB_2336820) for 1 h. After rinsing, sections were incubated in avidin-biotin-peroxidase complex (1:100; Vector Laboratories catalog #PK-6101, RRID:AB_2336820) for 1 h. Following a 0.1 M sodium acetate buffer rinse, sections were incubated with 0.05% diaminobenzidine intensified with Ni-glucose oxidase to yield a black product, mounted on slides, dried, dehydrated, and coverslipped.

To determine whether NK1R-expressing neurons co-expressed both Dab1 and the transcription factor *Lmx1b*, we used a tyramide signal amplification (TSA) immunofluorescence protocol for NK1R-Dab1-Lmx1b triple labeling. A rabbit antiserum to Dab1 (B3; 1:5000; generous gift of Dr. B. Howell, SUNY Upstate Medical University, Syracuse, NY; Howell et al., 1997) was used to detect Dab1 in slide-mounted 25-µm transverse sections. After a two-night incubation, biotinylated donkey anti-rabbit secondary IgG (1:1000, Jackson ImmunoResearch, catalog #711-065-152, RRID:AB_2340593) was applied to the sections, followed by streptavidin-conjugated horseradish peroxidase (1:1000, PerkinElmer catalog #NEL750001EA, RRID:AB_2617185). Then sections were incubated for 5 min in the tyramide reagent (1:100, TSA plus Cy5 kit, PerkinElmer, catalog #NEL745001KT). Before the second rabbit antiserum, sections were fixed for 15 min with 4% paraformaldehyde and washed repeatedly, followed by citric acid treatment as reported in Abadesco et al. (2014). Sections were then incubated in rabbit anti-NK1R overnight (1:20,000–1:30,000), followed by the steps listed above with the TSA Plus Fluorescein (1:200, PerkinElmer, catalog #NEL741B001KT). Next sections were placed in guinea pig anti-Lmx1b (1:20,000; generous gift of Dr. T. Müller and Dr. C. Birchmeier; Max Delbrück Center for Molecular Medicine, Berlin, Germany; RRID:AB_2314752; Müller et al., 2002), followed by donkey anti-guinea pig biotinylated antibodies (1:1000, Jackson ImmunoResearch, catalog #706-065-148, RRID:AB_2340451). After rinsing, sections were incubated in streptavidin-conjugated horseradish peroxidase, and followed by the tyramide reagent (1:150, TSA Plus Cy3, PerkinElmer, catalog #NEL744B001KT).

To determine the distribution of NK1R-expressing cells activated by noxious heat, we co-localized Fos and NK1Rs in male *dab1* mice. L4-5 spinal cords were cryosectioned at 20 µm and slide mounted. Fos immunohistochemistry was localized first with TSA. Sections were incubated with rabbit anti-Fos (1:25,000, Calbiochem/Millipore catalog #PC38, RRID:AB_2106755) overnight, rinsed in TNT buffer [0.1 M Tris (pH 7.5), 1.5% NaCl, and 0.05% Tween], and placed in biotinylated donkey anti-rabbit IgG (1:1000) for 1 h. After rinsing, streptavidin-conjugated horseradish peroxidase (1:100) was added, followed by fluorescein-labeled tyramide for 8 min (1:50, TSA Plus Fluorescein). After citric acid treatment, sections were incubated with rabbit anti-NK1R (1:15000, Sigma) using standard techniques with a donkey anti-rabbit Alexa Fluor 594 secondary (1:800, Jackson ImmunoResearch, catalog #711-585-152, RRID:AB_2340621). To compare the levels of NK1Rs after SSP-SAP treatment, free-floating 40-µm cervical and lumbar spinal cords were incubated with anti-NK1R (1:25,000), and localized with TSA techniques as described above (TSA Plus Cy3).

### Experimental design and statistical analyses

The initial analysis of NK1R-immunoreactive neurons examined one dorsal horn from ∼25 sections/mouse in 10 *Reln* and eight *dab1* male or female mice, evenly divided by wild-type and mutant genotypes. Sections were photographed (Olympus AX70) and images were used to record the NK1R-expressing neurons identified with the microscope (40×). Dorsal horns were divided into Laminae I–II, III–IV, and V–VI plus the LSN (as in Wang et al., 2012) and NK1R-expressing cells with one or more processes were counted. Values across sections from the same mouse and location were averaged to generate one value per mouse and location. Means between wild-type and mutant mice were compared at each location using the *post hoc t* comparisons under a two-way (genotype × location) repeated measures (mixed-random effects) ANOVA model (JMP Software version 13, SAS Institute). The Fisher LSD criterion was used to control for multiple comparisons under this model.

To quantify subtypes of NK1R-expressing neurons that co-express Dab1 and/or Lmx1b in Laminae I–II, we analyzed six dorsal horn hemisections/mouse from six *Reln^+/+^* and six *Reln^-/-^* mice by collecting confocal images at 40× with a Zeiss LSM800 microscope. Confocal slices (3 µm) were used to identify subtypes of NK1R cells: NK1R-only, NK1R-Dab1, NK1R-Lmx1b, and NK1R-Dab1-Lmx1b neurons. Means were compared between the two genotypes across four NK1R subsets using a 2 × 4 repeated measure ANOVA model as above. The *p* values for comparing means of total NK1R neurons between the two genotypes were computed using a *t* test.

The Fos/NK1R analysis was conducted on three to five dorsal horns per mouse in six *dab1^+/+^* and six *dab1^-/-^* male mice. Four to five 25× confocal images were acquired (Zeiss LSM 510) for the Laminae I–II and LSN analyses. To image a total of 6- to 8-µm/dorsal horn, we collected serial 3 µm slices. Cell counts were conducted from 3-D reconstructions of each confocal slice with Neurolucida (v10; MicroBrightField RRID:nif-0000-10294). Double-labeled neurons per area per mouse were averaged and means between wild-type and mutant mice were compared using a two-way (genotype × cell type) repeated measures ANOVA and *post hoc t* test.

The *dab1* (seven wild-type and seven mutant) mice were examined using the Hargreaves and von Frey tests of thermal heat and mechanical sensitivity, respectively, before and after SSP-SAP treatment (one mutant died before retest). Means from the Hargreaves test were compared with a two-way (genotype × treatment) repeated measure ANOVA as above. The Mann–Whitney test was used to compare means of baseline and post SSP-SAP mechanical thresholds from the von Frey in the two genotypes, as these data do not have a normal distribution. The Wilcoxon matched pairs signed-rank test evaluated differences of treatments within genotypes (Sigma Plot 12, Systat Software Inc.).

As a final experiment, we tested *dab1* mice on the hot plate test to extend our Hargreaves analyses and better interpret the heat responses of the mutant mice. Videos were analyzed blind to temperature, but the reeling behavior of mutants precludes blinding to genotype. Results of the three trials at each temperature were averaged for each of the 11 *dab1^+/+^* and 11 *dab1^-/-^* female mice tested. Means were compared between *dab1^+/+^* and *dab1^-/-^* mice with a two-way (genotype × temperature) repeated measures ANOVA, followed by a *post hoc t* test.

## Results

### Compared to *Reln^-/-^*, *Reln^+/+^* mice have fewer NK1R-expressing neurons in Laminae I–II and more in the LSN

**Figure 1.**
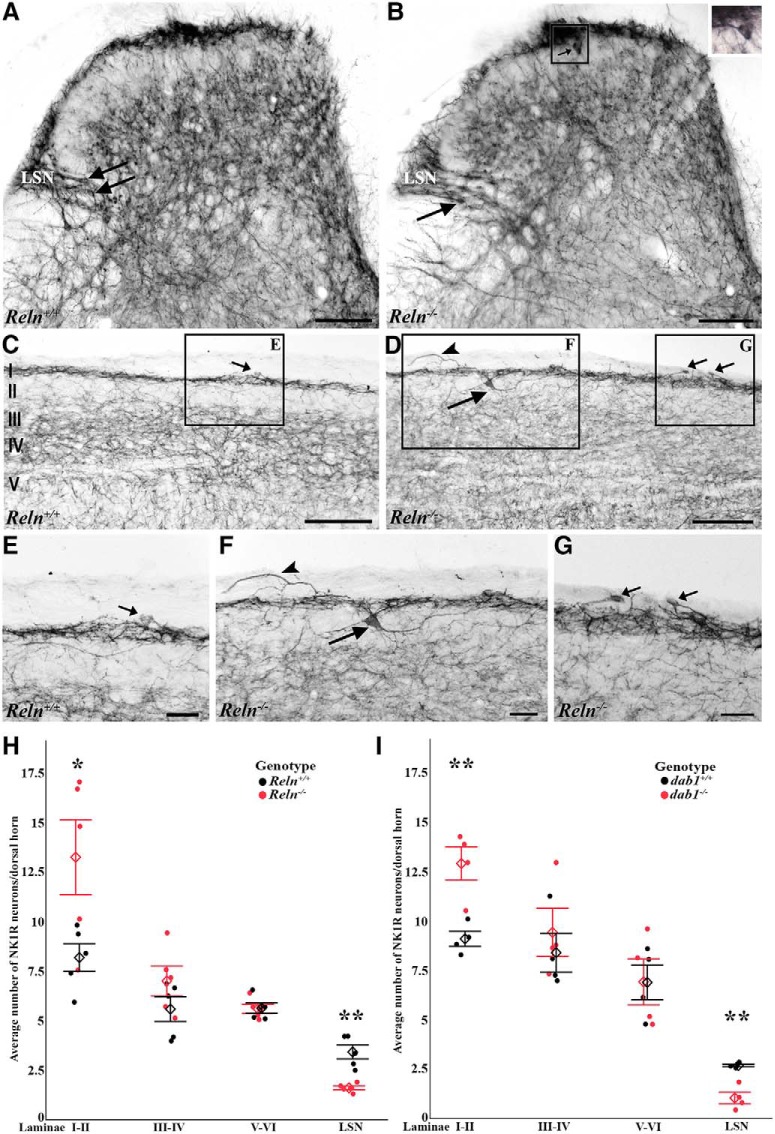
Fewer neurons express NK1Rs in *Reln^+/+^* (***A***, ***C***, ***E***) than *Reln^-/-^* (***B***, ***D***, ***F***, ***G***) Laminae I–II. ***A***, ***B***, Transverse sections show a similar pattern of NK1R expression, but the *Reln^-/-^* has a prominent labeled neuron in Lamina II (***B***, small arrow, enlarged in inset), not usually found in *Reln^+/+^* dorsal horn (***A***). The lateral spinal nucleus (LSN) displays substantial NK1R expression, and a few cells (***A***, ***B***, large arrows). ***C–G***, Low magnification images of sagittal sections of *Reln^+/+^* (***C***) and *Reln^-/-^* (***D***) dorsal horns. Boxed areas with NK1R-bearing neurons (arrows) and processes (arrowheads) are enlarged in E-G. ***H***, ***I***, Black and red circles represent wild-type and mutant mice, respectively. Open diamonds display group mean ± SEM values. Average number of NK1R neurons analyzed by dorsal horn Laminae in *Reln* (***H***; Laminae I–II, *p = 0.0359; LSN, ** *p* = 0.0011) and *Dab1* mice (***I***; Laminae I–II, **p = 0.0061; LSN, ** *p* = 0.0016). Scale bars = 100 µm (***A***, ***B***, ***E–G***), 200 µm (***C***, ***D***).

Previously, we described mispositioned NK1R-Dab1-expressing neurons in *Reln^-/-^* and *Apoer2^-/-^/Vldlr^-/-^* mice at the Laminae II–III border (Villeda et al., 2006; Akopians et al., 2008). Here, we ask whether loss of the Reelin-Dab1-signaling pathway also altered the number of neurons bearing NK1Rs in the superficial dorsal horn. Based on analyses of lower lumbar dorsal horn sections (Fig. 1*A*,*B*), we found that, on average, there are significantly fewer NK1R-expressing neurons in *Reln^+/+^*than in *Reln^-/-^* Laminae I–II (*Reln^+/+^* 8.2 neurons ±0.7 SEM, *n* = 5 mice; *Reln^-/-^* 13.2 ± 1.9, *n* = 5, *p* = 0.0359; Fig. 1*H*). We found no differences between the numbers of NK1R-expressing neurons in Laminae III–VI (Fig. 1*H*). The differences in NK1R-expressing neurons in *Reln^+/+^*and *Reln^-/-^* mice are readily appreciated in sagittal sections of the dorsal horn (Fig. 1*C–G*). Occasionally we recorded large, ectopic NK1R-expressing neurons in Lamina II of *Reln^-/-^* mice (Fig. 1*D*,*F*, large arrows) and found more NK1R-immunoreactive processes in the white matter dorsal to Lamina I in mutant than in wild-type mice (Fig. 1*D*,*F*, arrowheads). In distinct contrast, the *Reln^+/+^*LSN contains twice as many NK1R-expressing neurons as does *Reln^-/-^*LSN (*Reln^+/+^* 3.4 ± 0.4, *n* = 5; *Reln^-/-^* 1.6 ± 0.1, *n* = 5, *p* = 0.0011; Fig. 1*H*), consistent with a loss of ∼50% of total LSN neurons in mutants (Villeda et al., 2006). Compared to *Reln^+/+^,* therefore, there are more NK1R-expressing neurons in *Reln^-/-^*Laminae I–II and fewer in the LSN.

### The d*ab1^-/-^*mice also have more NK1R-expressing neurons in Laminae I–II and fewer in the LSN than *dab1^+/+^* mice

To verify that the Reln-Dab1 signaling pathway is critical to the incorrect positioning of neurons expressing NK1Rs, we next analyzed the number and distribution of neurons that express NK1Rs in *dab1* dorsal horns. In *dab1^+/+^* Laminae I–II, we found fewer cells with NK1Rs than in *dab1^-/-^* mice (*dab1^+/+^* 9.1 ± 0.4, *n* = 4 mice; *dab1^-/-^* 12.9 ± 0.8, *n* = 4; *p* = 0.0061; Fig. 1*I*). There were no differences between genotypes in Laminae III–VI (Fig. 1*I*). We also found more than twice the number of NK1R-expressing neurons in the LSN of *dab1^+/+^* compared to *dab1^-/-^* LSN (*dab1^+/+^*2.7 ± 0.06, *n* = 4; *dab1^-/-^* 1.0 ± 0.3, *n* = 4; *p* = 0.0016; Fig. 1*I*). As positioning errors of NK1R-imunoreactive neurons are comparable in the superficial dorsal horn and LSN of *Reln^-/-^*and *dab1^-/-^* mice, we suggest that these errors are secondary to the disruption of the canonical Reln-Dab1 signaling pathway.

### Many NK1R-Dab1 neurons also express Lmx1b and thus are excitatory

**Figure 2.**
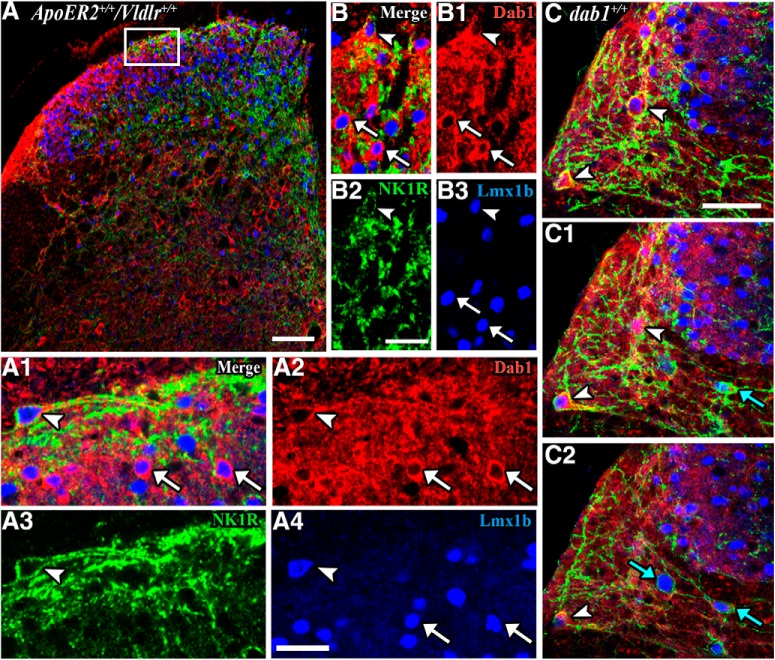
NK1R-expressing neurons co-express Dab1 and Lmx1b. Confocal slices (1–3 µm thick) show expression of Dab1 (red cytoplasm), NK1R (green receptors), and Lmx1b (blue nuclei) in wild-type (*ApoER2^+/+^/Vldlr^+/+^* or *dab1^+/+^*) dorsal horn. Medial is oriented to the right in this and subsequent figures. ***A***, Boxed area in the dorsal horn hemisection (***A***) is enlarged in ***A1–A4***. The large NK1R-expressing neuron in Lamina I (***A1***, ***A3***, arrowheads) co-expresses Dab1 (***A2***, arrowhead) and Lmx1b (***A4***, arrowhead). Dab1-Lmx1b neurons (***A1***, ***A2***, ***A4***; white arrows) are obvious in Lamina II. ***B***, A small NK1R-expressing cell in Lamina I (***B***, ***B2***, white arrowheads) also expresses Dab1 (***B1***, arrowhead) and Lmx1b (***B3***, arrowhead). Dab1-Lmx1b neurons (***B***, ***B1***, ***B3***, white arrows) are found in Lamina II. ***C***, Serial confocal images of the lateral spinal nucleus illustrate two large NK1R-Dab1-Lmx1b neurons (***C–C2***; arrowheads) and two NK1R-Lmx1b neurons (***C1***, ***C2***; blue arrows). Scale bars = 100 µm (***A***), 25 µm (***A1–A4***, ***B–B3***), 50 µm (***C–C2***).

Recently we reported that 70% of Dab1-expressing neurons in the superficial dorsal horn co-express the transcription factor Lmx1b. Additionally, noxious heat and mechanical stimulation induced Fos expression in Dab1-Lmx1b-labeled neurons in the superficial dorsal horn and lateral reticulated area (Yvone et al., 2017). Now we ask whether the NK1R-Dab1-expressing neurons also express Lmx1b or if they comprise a separate population of Dab1 neurons. In wild-type Lamina I, we found large NK1R-expressing neurons that co-express both Dab1 and Lmx1b (Fig. 2*A–A4*, arrowheads) and resemble reported NK1R-bearing projections neurons (Marshall et al., 1996; Todd et al., 2002; Spike et al., 2003). Examples of Dab1-Lmx1b neurons that are not associated with NK1Rs are common (Fig. 2*A1*,*A2*,*A4*,*B*,*B1*,*B3*, white arrows), as are Lmx1b-labeled neurons surrounded by NK1Rs. In addition, there are smaller Dab1 neurons that express NK1Rs found in Laminae I–II, some of which have Lmx1b-labeled nuclei (Fig. 2*B–B3*, arrowheads). Serial confocal images of wild-type LSN illustrate several triple-labeled cells (Fig. 2*C–C2*, arrowheads) and also double-labeled NK1R-Lmx1b neurons (Fig. 2*C1*,*C2*, blue arrows). These findings establish that superficial dorsal horn NK1R-Dab1 neurons are indeed excitatory (Littlewood et al., 1995; Szabo et al., 2015) and suggest that NK1R-Dab1-expressing neurons may include both projection neurons and interneurons (Cheunsuang and Morris, 2000; Al Ghamdi et al., 2009).

**Figure 3.**
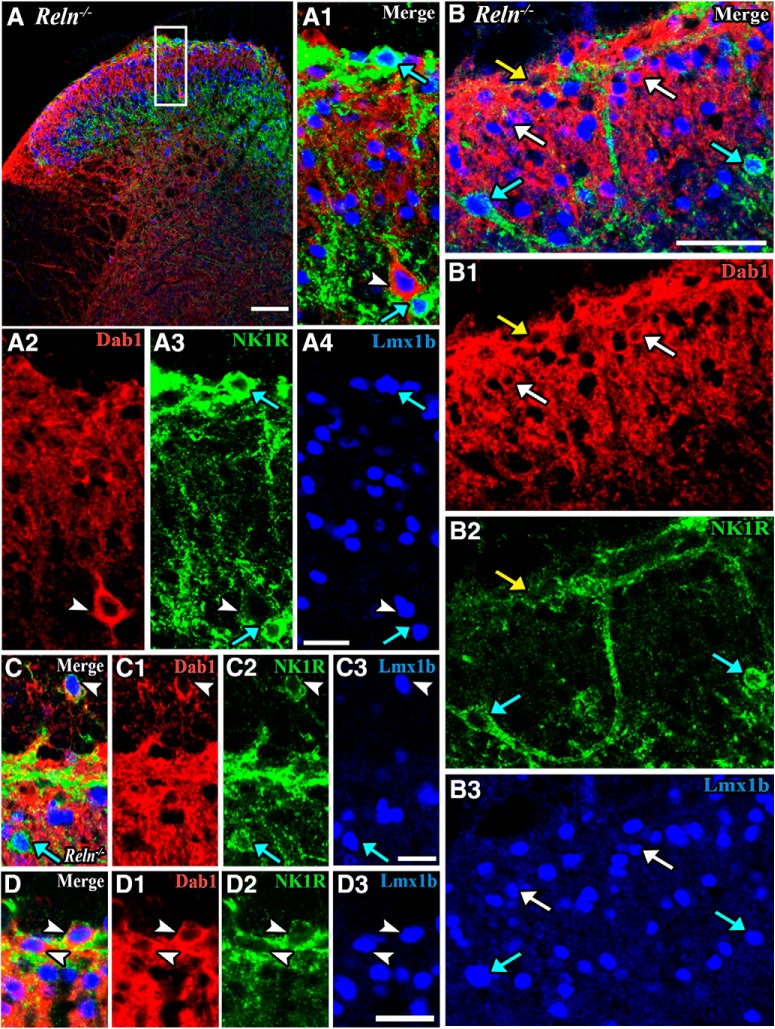
Evidence of mispositioned NK1R-expressing neurons in *Reln^-/-^*Laminae I–II. Triple immunofluorescent images show Dab1 (red cytoplasm), NK1R (green receptors), and Lmx1b (blue nuclei) expression in *Reln^-/-^* superficial dorsal horn. ***A***, Box in ***A*** is enlarged in ***A1–A4***. A large Dab1-Lmx1b neuron is surrounded by NK1Rs and found near the Laminae II–III border (***A1–A4***, white arrowheads). Two NK1R-Lmx1b neurons (***A1***, ***A3***, ***A4***, blue arrows) are shown. ***B***, Several NK1R-Lmx1b neurons are observed in *Reln^-/-^* Lamina II (***B***, ***B2***, ***B3***, blue arrows) and one of them appears mispositioned with an unusual dendritic process (***B***, ***B2***, ***B3***, left blue arrows). A Dab1 neuron that displays only NK1Rs is found in Lamina I (***B***, ***B1***, ***B2***, yellow arrows). Several Dab1-Lmx1b neurons are also present (***B***, ***B1***, ***B3***, white arrows). ***C***, Triple-labeled cell is mispositioned in the dorsal white matter of *Reln^-/-^*mouse (***C–C3***, arrowheads). Double-labeled NK1R-Lmx1b neurons are marked by blue arrows (***C***, ***C2***, ***C3***). ***D***, Small neurons that co-express NK1R-Dab1-Lmx1b are found in *Reln^-/-^*Lamina I (***D–D3***, arrowheads). Scale bars = 100 µm (***A***), 25 µm (***A1–A4***, ***C–C3***, ***D–D3***), 50 µm (***B–B3***).

In the *Reln^-/-^* Laminae I–II a number of Lmx1b neurons are covered with NK1Rs (Fig. 3*A*,*A1*,*A3*,*A4*,*B*,*B2*,*B3*,*C*,*C2*,*C3*, blue arrows). Additionally, in *Reln^-/-^*dorsal horn we observed NK1R-Dab1-Lmx1b neurons that are distinctly mispositioned in the dorsal white matter (Fig. 3*C–C3*, arrowheads). In mutant mice, we also recorded an ectopic, large, triple-labeled neuron near the border between Laminae II–III (Fig. 3*A–A4*, arrowheads), where NK1R neurons are rarely found in wild-type mice. We also observed a large NK1R-Lmx1b neuron in *Reln^-/-^*Lamina II. This neuron emitted a long, uncharacteristically-oriented dendrite (Fig. 3*B*,*B2*,*B3*, blue arrows) reminiscent of NK1R projection neurons in Lamina III (Todd et al., 2000). Other NK1R-Dab1-expressing neurons in Lamina I are small and may or may not have Lmx1b nuclei (Fig. 3*D–D3*, arrowheads).

**Figure 4.**
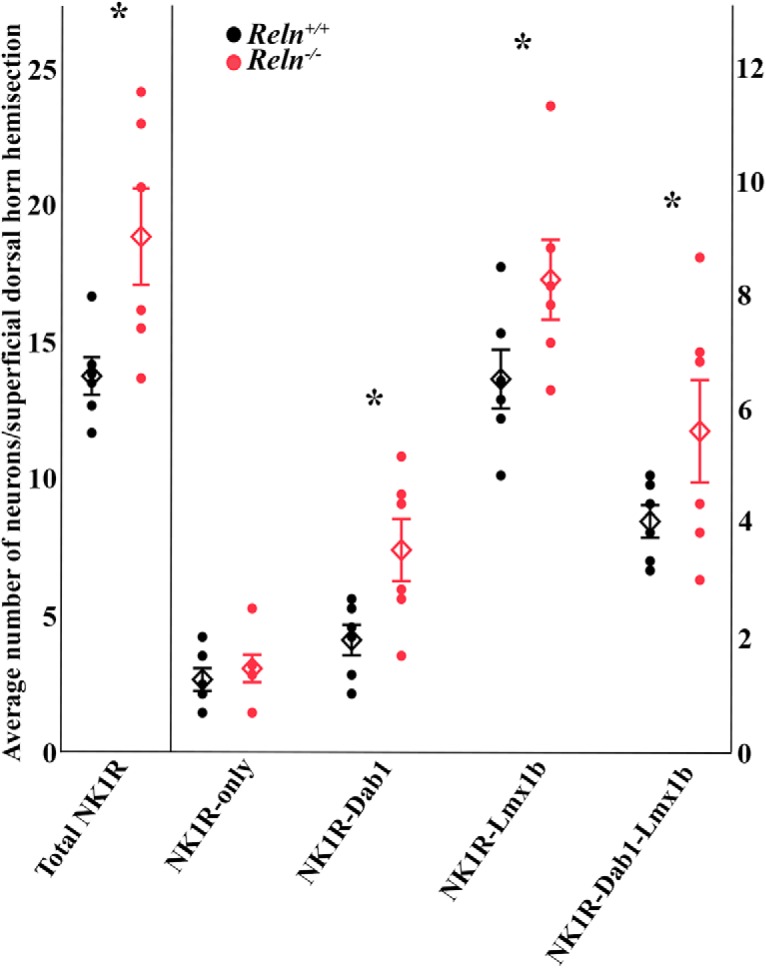
Multiple NK1R subtypes in Laminae I–II express Dab1 and/or Lmx1b. Graphical representation of the average number of NK1R-expressing neurons in Laminae I–II per hemisection (3-µm slice). Circles represent individual mice and diamonds mark group mean ± SEM values. Greater numbers of total NK1R-expressing cells shown in *Reln^-/-^* (red circles) versus *Reln^+/+^* (black circles) Laminae I–II (left side, *p = 0.0227). Three of the four NK1R subtypes are more numerous in mutant than wild-type mice (right side, NK1R-Dab1 neurons; *p = 0.0368; NK1R-Lmx1b neurons, *p = 0.0219; NK1R-Dab1-Lmx1b neurons, *p = 0.0368).

To determine whether the numbers and subtypes of the adult NK1R-expressing neurons differ between wild-type and mutant mice, we quantified these neurons in Laminae I–II. *Reln^-/-^*mice had greater numbers of total NK1R-expressing neurons in the superficial dorsal horn compared to *Reln^+/+^*mice (*Reln^+/+^*13.8 ± 0.7 neurons/hemisection, *n* = 6 mice; *Reln^-/-^* 18.9 ± 1.8, *n* = 6; *p* = 0.0227; Fig. 4). The small number of NK1R-only neurons did not differ (Fig. 4), but the other three NK1R subsets had greater numbers of NK1R neurons in *Reln^-/-^* versus *Reln^+/+^*mice. Figure 4 illustrates the means of NK1R-Dab1 neurons (*Reln^+/+^*1.9 ± 0.3, *n* = 6; *Reln^-/-^*3.5 ± 0.5, *n* = 6; *p* = 0.0368), NK1R-Lmx1b cells (*Reln^+/+^*6.5 ± 0.5; *Reln^-/-^* 8.3 ± 0.7; *p* = 0.0219), and the triple-labeled NK1R-Dab1-Lmx1b neurons (*Reln^+/+^*4.0 ± 0.3; *Reln^-/-^*5.6 ± 0.9; *p* = 0.0368) and shows that they differ between genotypes. Almost 75% of our wild-type and mutant NK1R neurons co-express Lmx1b, and the remainder may also be excitatory (Littlewood et al., 1995; Szabo et al., 2015). In addition, these analyses found that 43.5% (wild type) and 48% (mutant) of these NK1R neurons co-express Dab1 and thus would be influenced by the loss of Reln signaling.

### The pattern of noxious heat-induced Fos expression in *dab1^+/+^* and *dab1^-/-^* mice parallels the distribution of NK1R-expressing neurons

**Figure 5.**
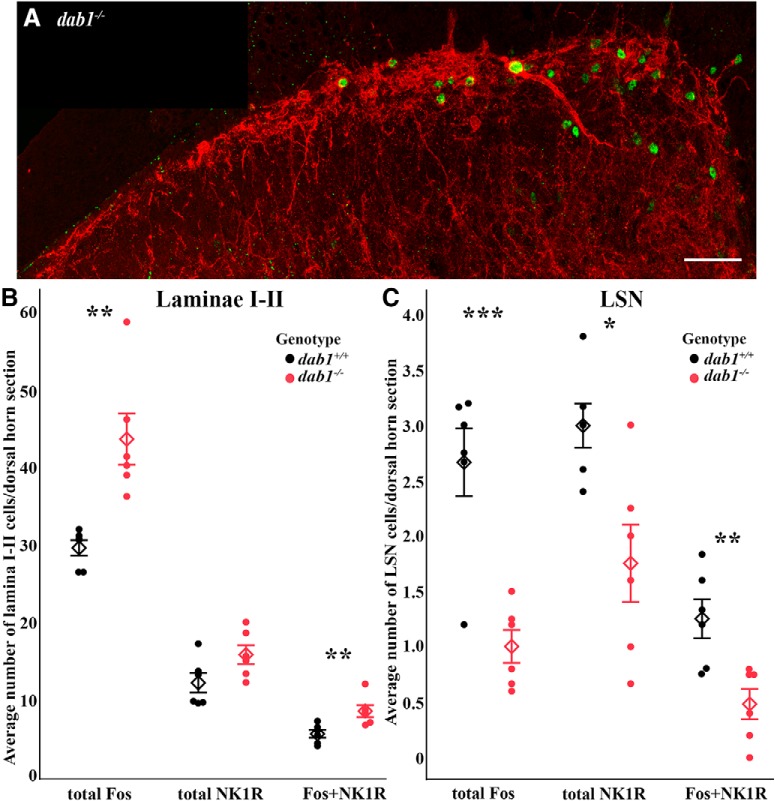
Noxious heat induces Fos expression in *dab1^+/+^* and *dab1^-/-^*dorsal horn. ***A***, An example of the confocal images used to analyze NK1R-Fos expression in *dab1^-/-^* Laminae I–II has a large misplaced NK1R-Fos-labeled cell body in Lamina I. ***B***, After thermal stimulation *dab1^+/+^*mice (black circles) have fewer Fos-expressing neurons in Lamina I–II than *dab1^-/-^* mice (red circles, **p = 0.0028). Total NK1R-expressing neurons did not vary by genotype. NK1Rs and Fos-expressing neurons in Laminae I–II were less numerous in *dab1^+/+^*than in *dab1^-/-^*mice (**p = 0.0097). ***C***, After heat stimulation, the total number of Fos-labeled neurons in the LSN was higher in *dab1^+/+^* than *dab1^-/-^* mice (***p = 0.00064). The total NK1R- and NK1R-Fos-expressing neurons also were more numerous in *dab1^+/+^*than *dab1^-/-^* LSN (*p = 0.0113, **p = 0.0062, respectively). Circles (*dab1^+/+^* are black and *dab1^-/-^* are red) represent individual mice and diamonds show group mean ± SEM values. Scale bar = 50 µm (***A***).

Wang et al. (2012) reported that after noxious heat stimulation, wild-type (*Reln^+/+^*and *dab1^+/+^*) mice had many less Fos-labeled-immunoreactive neurons in Laminae I–II, but greater numbers in the LSN than are found in mutant (*Reln^-/-^* and *dab1^-/-^*) mice. Here, we asked whether the increased numbers of NK1R neurons co-express Fos in *dab1^-/-^* compared to *dab1^+/+^* superficial dorsal horn and conversely, if decreased numbers are found in *dab1^-/-^* than *dab1^+/+^* LSN (Fig. 5). Based on an analysis using 3-D reconstructions of confocal images of Laminae I–II (Fig. 5*A*) and the adjacent LSN, we found that the *dab1^+/+^*superficial dorsal horn had on average 5.6 ± 0.5 neurons/section (i.e., a 6- to 8-µm confocal slice from six mice) that co-expressed Fos and NK1Rs. By comparison, *dab1^-/-^* mice contained 8.5 ± 0.8 double-labeled cells/hemisection (*n* = 6, *p* = 0.0097; Fig. 5*B*). Figure 5*A* illustrates an example of a large, ectopic NK1R-Fos-expressing neuron in *dab1^-/-^* Lamina I with a long atypical dendrite that projects into Lamina II. Next, we compared the percentage of Fos-expressing neurons that co-express NK1Rs after heat stimulation: 19% of total Fos cells in Laminae I–II of both *dab1* genotypes co-expressed NK1Rs. This is consistent with our findings that *dab1^-/-^* mice have greater numbers of Fos and more Fos-NK1R-expressing cells than *dab1^+/+^* mice. Of the total number of NK1R-expressing neurons we found that 46% of *dab1^+/+^*and 54% of *dab1^-/-^*Laminae I–II neurons co-expressed Fos (Fig. 5*B*), a finding consistent with our hypothesis that noxious heat activates higher numbers of NK1R-expressing neurons, some of which must be mispositioned.

In the LSN, we determined that the 6 *dab1^+/+^* mice had 1.3 ± 0.2 Fos-immunoreactive NK1R neurons per section compared to only 0.5 ± 0.1 double-labeled neurons in the 6 *dab1^-/-^* mice (*p* = 0.0062; Fig. 5*C*). The percentage of total Fos-immunoreactive cells that bear NK1Rs in the LSN again is similar in both genotypes, with 49% of Fos-labeled cells being double-labeled. Of the total NK1R-immunoreactive neurons, 42% of *dab1^+/+^*and 27% of *dab1^-/-^*LSN were double-labeled after heat stimulation. These percentages reflect the overall loss of neurons in the mutant LSN (Villeda et al., 2006; Akopians et al., 2008; Wang et al., 2012).

**Figure 6.**
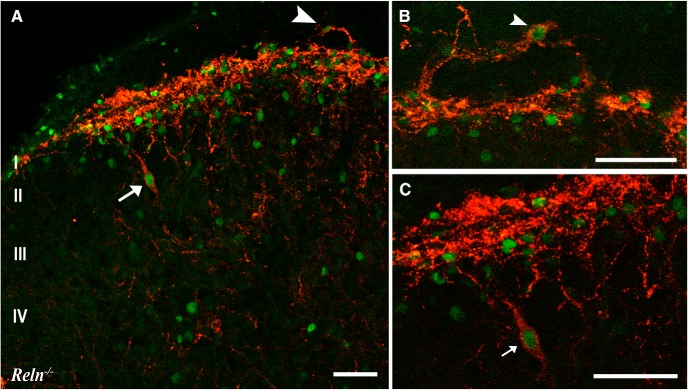
After thermal stimulation ectopic NK1R neurons express Fos in *Reln^-/-^* dorsal horn. ***A***, NK1Rs (red) are highly expressed in Lamina I. Mispositioned NK1R-bearing neurons express Fos (green) near the Laminae II–III border (arrow) and in the dorsal white matter (arrowhead). ***B***, ***C***, Neurons that co-express NK1Rs and Fos in the dorsal white matter are enlarged from a nearby section (***B***, arrowhead), whereas the mispositioned neuron near the Laminae II–III border was enlarged from ***A*** (***C***, arrow). Scale bars = 50 µm (***A–C***).

We also observed that heat stimulation activated mispositioned NK1R-expressing cells in *Reln^-/-^*superficial dorsal horn. For example, Figure 6*A*,*C* illustrates a Fos-immunoreactive NK1R-expressing cell near the border of Laminae II–III, in a similar position to the large NK1R-Dab1-Lmx1b neuron mispositioned in *Reln^-/-^* mice (Fig. 3*A–A4*). The dorsal white matter in the *Reln^-/-^* mice also contained NK1R-immunoreactive processes as well as double-labeled Fos-NK1R-expressing neurons (Fig. 6*A*,*B*), and represents another area where ectopic NK1R-expressing neurons are found (Fig. 3*C–C3*). Together, these findings demonstrate that mispositioned NK1R-expressing neurons do indeed respond to noxious heat stimulation in both the *Reln^-/-^* and *dab1^-/-^*dorsal horns.

### Ablation of NK1R-expressing neurons eliminates the heat hypersensitivity of *dab1^-/-^* mice

**Figure 7.**
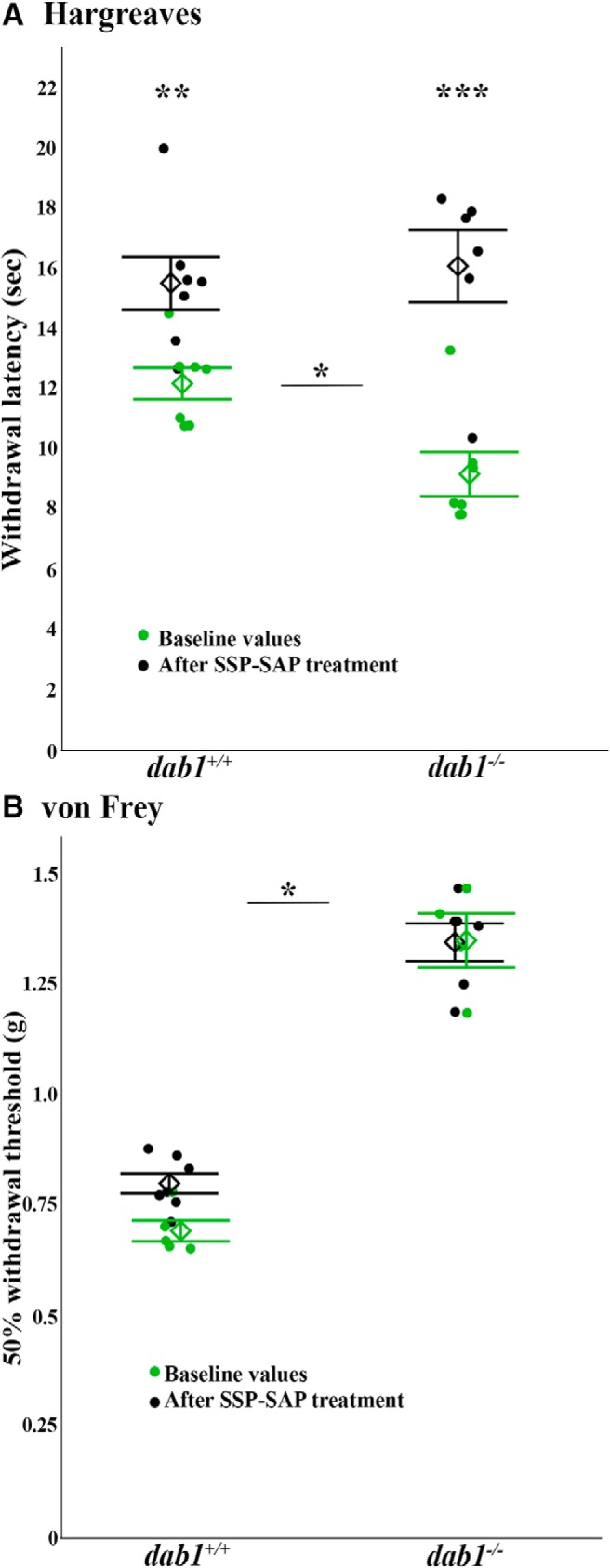
SSP-SAP eliminates the thermal hypersensitivity of *dab1^-/-^* mice. ***A***, Hargreaves test results illustrate that baseline values (green circles) for the *dab1^+/+^* and *dab1^-/-^* withdrawal responses differ by genotype (*p = 0.0245). After SSP-SAP treatment (black circles), the *dab1^+/+^* (**p = 0.0069), and *dab1^-/-^* (***p < 0.0001) responses were reduced compared to their respective baseline values. ***B***, The von Frey responses in *dab1^+/+^* and *dab1^-/-^* mice do not change with SSP-SAP treatment. Compared to the *dab1^+/+^*, *dab1^-/-^*mice have much slower responses both before (*p = 0.0159) and after SSP-SAP treatment (*p = 0.0034). Circles represent individual mice and diamonds show group mean ± SEM.

As first reported (Akopians et al., 2008), we again found that the *dab1^-/-^* mice have a reduced latency to withdraw from noxious heat in the Hargreaves test. For the present cohort of mice, the baseline withdrawal average latency for *dab1^+/+^*mice was 12.2 ± 0.9 s versus *dab1^-/-^* mice at 9.3 ± 0.8 s (*p* = 0.025, *n* = 7 mice/genotype; Fig. 7*A*). After establishing baseline responsiveness, all mice received a single lumbar intrathecal injection of SSP-SAP and one month later we retested withdrawal latencies. Compared to baseline values, both *dab1^+/+^* (15.7 ± 0.82 s, *n* = 7, *p* = 0.0069) and *dab1^-/-^* mice (*dab1^-/-^* 15.83 ± 0.88 s, *n* = 6; *p* = 0.0001; Fig. 7*A*) exhibited increases in their withdrawal latency, but most importantly, increases were significantly greater in the *dab1^-/-^*mice. In addition, the mean values of both genotypes did not differ after the SSP-SAP treatment (Fig. 7*A*). These results show that mispositioned NK1R-expressing neurons are required for the thermal (heat) hypersensitivity of *dab1^-/-^* mice. We presume that our finding of equivalent post treatment latencies reflects the fact that both correctly and incorrectly positioned NK1R-expressing neurons were ablated in the *dab1^+/+^* and *dab1^-/-^*mice.

To determine the modality specificity of the behavioral changes observed, we also asked whether the loss of superficially located NK1Rs altered mechanical sensitivity. The baseline testing data replicated our previous results (Akopians et al., 2008) of increased thresholds in the mutant mice. The initial mean 50% threshold for the *dab1^+/+^*mice was 0.69 ± 0.02 g (*n* = 7) compared to *dab1^-/-^* mice at 1.35 ± 0.06 g (*n* = 7; *p* = 0.0159; Fig.7*B*). One month following SSP-SAP treatment, the withdrawal thresholds also differed between genotypes (*dab1^+/+^* 0.80 ± 0.02, *n* = 7; *dab1^-/-^*1.35 ± 0.04; *n* = 6; *p* = 0.0034; Fig 7*B*). The mean 50% threshold for both *dab1^+/+^* (0.80 ± 0.02 g, *n* = 7 *p* = 0.063) and *dab1^-/-^*mice (1.35 ± 0.04 g, *p* = 0.999; *n* = 6) after treatment, however, did not vary from their baseline values (Fig. 7*B*). We conclude that ablation of NK1R-expressing neurons does not alter the profound mechanical insensitivity seen in *dab1^-/-^*mice. It follows that the increased mechanical withdrawal thresholds are likely due to other groups of mispositioned neurons in the superficial dorsal horns of the *dab1^-/-^*mice.

**Figure 8.**
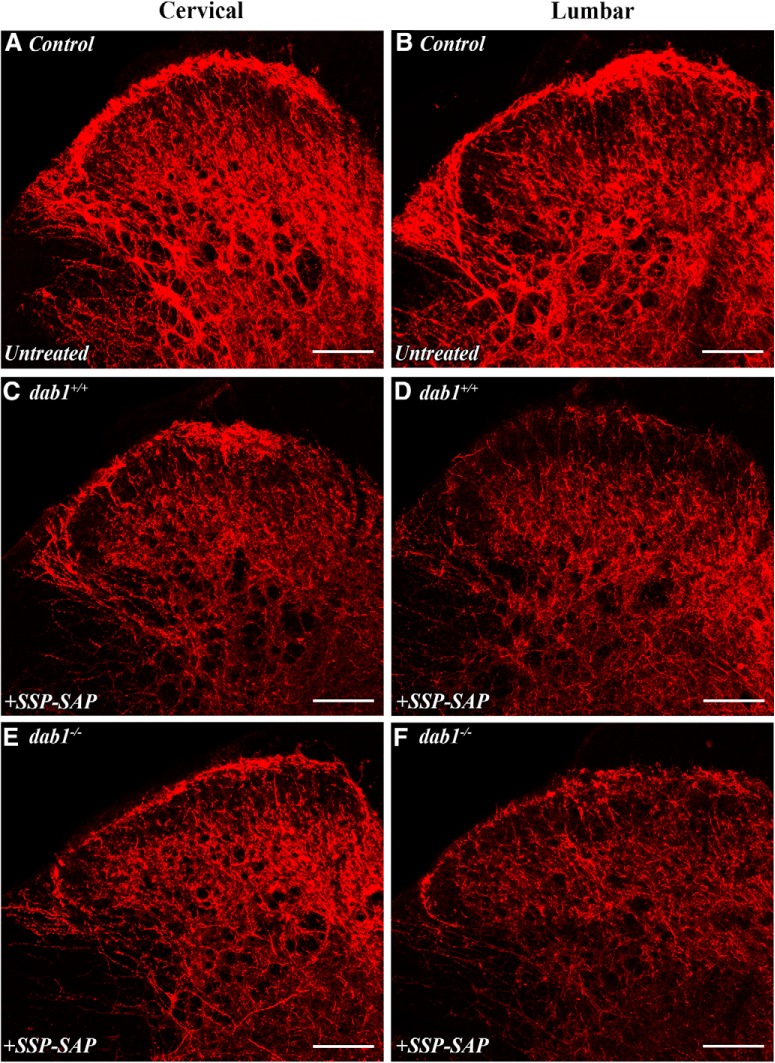
NK1R expression is greatly reduced by intrathecal SSP-SAP. All sections were incubated together in the same immunoreagents and confocal images taken at the same settings. ***A***, ***B***, Control cervical (***A***) and lumbar (***B***) sections have high NK1R expression levels in Lamina I and the LSN. ***C***, ***D***, SSP-SAP-treated *dab1^+/+^* cervical (***C***) section has higher NK1R expression in Lamina I and LSN than the lumbar section (***D***, target of the intrathecal injection). ***E***, ***F***, Levels of NK1R-expression are higher in the *dab1^-/-^*cervical Lamina I (***E***) than in the lumbar section (***F***). The lumbar LSN has particularly low levels of NK1R immunoreactivity in SSP-SAP treated mice. Scale bars = 100 µm (***A–F***).

Importantly, to document the ablation of NK1R neurons after SSP-SAP, we compared the level of NK1R immunoreactivity in lumbar (directly targeted) and cervical sections from ablated mice to that in untreated spinal cord sections. Figure 8 shows representative levels of NK1R expression in cervical and lumbar spinal cord from untreated mice and after SSP-SAP treatment. NK1R expression was noticeably decreased in lumbar Lamina I and LSN after SSP-SAP (Fig. 8*D*,*F*) compared to the same areas in untreated mice (Fig. 8*B*). Somewhat unexpectedly, NK1R expression in cervical enlargement sections of treated mice was also reduced (Fig. 8*C*,*E*) compared to untreated mice (Fig. 8*A*), but to a lesser extent than in lumbar cord. This presumably resulted from rostral diffusion of the SSP-SAP reagent. Together, these results confirm the efficacy of the SSP-SAP treatment and suggest that the mispositioned NK1Rs in *dab1^-/-^* mice are responsible for the thermal hyperalgesia.

**Figure 9.**
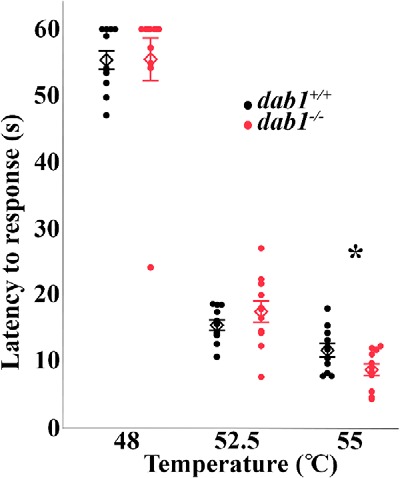
*dab1^-/-^* mice respond to 55°C stimulation on a hot plate faster than *dab1^+/+^* mice. The hot plate test examined latency responses to 48°C, 52.5°C, and 55°C. Differences between genotypes were limited to 55°C, with *dab1^-/-^* responding more quickly than *dab1^+/+^* mice (*p = 0.0395). Circles (black for *dab1^+/+^* and red for *dab1^-/-^*) represent individual mice and diamonds mark group mean ± SEM.

Finally, we asked whether the heat hypersensitivity revealed using the Hargreaves test, which likely reflects increased excitability of circuits that underlie a spinally-mediated withdrawal reflex response, is also manifest in the hot plate test, which we believe involves supraspinal as well as spinal cord processing of “pain” messages. Consistent with our hypothesis, Figure 9 shows that despite the coordination difficulties characteristic of these mutant mice, there was a significant decrease of the latency to respond to the 55°C hot plate stimulation (*dab1^+/+^* 11.5 ± 1.01 s, *n* = 11; *dab1^-/-^* 8.6 ± 0.87 s, *n* = 11, *p* = 0.0395).

## Discussion

This study addresses the basis for the thermal hyperalgesia that occurs in Reln-signaling mutants. First, we found increased numbers of neurons that express NK1Rs in dorsal horn Laminae I–II in *Reln^-/-^* and *dab1^-/-^* and fewer in mutant LSN compared to their respective wild-type mice. We then showed that some NK1R neurons co-express Dab1 and Lmx1b, confirming their excitatory phenotype (Cheng et al., 2004, 2005; Dai et al., 2008; Szabo et al., 2015; Yvone et al., 2017). Next, we showed that noxious heat stimulation induced Fos-expression in significantly greater numbers of NK1R-immunoreactive neurons in *dab1^-/-^* Laminae I–II and fewer in *dab1^-/-^*LSN than in *dab1^+/+^* mice. Importantly, based on their incorrect position in Lamina I, in the dorsal white matter, or near the Laminae II–III border, we conclude that at least some of the incorrectly positioned NK1R neurons in *Reln^-/-^* and *dab1^-/-^* mice express Fos in response to noxious heat stimulation, which establishes that these neurons are functional. By ablating the superficial NK1R-bearing neurons with intrathecal SSP-SAP, we eliminated the thermal hyperalgesia of the *dab1^-/-^*mice, without altering their mechanical sensitivity. We conclude that misplaced NK1R-expressing neurons, specifically those that populate the superficial laminae of the dorsal horn, underlie the heat hypersensitivity that characterizes the Reln-signaling pathway mutants.

NK1R-expressing neurons constitute the majority of Lamina I projection neurons, are excitatory, and transmit moderate to intense pain-provoking information to supraspinal, brainstem and thalamic targets (Littlewood et al., 1995; Marshall et al., 1996; Todd et al., 2005; Todd, 2010). Another population of more ventrally located NK1R-expressing projection neurons has dorsally-directed dendrites that intermingle with the Lamina I projection neurons (Brown et al., 1995; Todd, 2010). Both populations of NK1R projection neurons are targeted by transient receptor potential vanilloid 1 (TRPV1)-expressing primary afferent fibers that respond to noxious heat stimulation. In fact, because intrathecal capsaicin-induced ablation of TRPV1 terminals completely abrogated both acute noxious heat-evoked behaviors, as well as the heat hyperalgesia that occurs in the setting of tissue injury, we previously concluded that the TRPV1 afferents provide the predominant, and likely the exclusive noxious heat input to the dorsal horn (Cavanaugh et al., 2009).

We do not believe, however, that an increased afferent input to the altered dorsal horn circuits underlies the heat hypersensitivity of these mutant mice. In fact, we observed neither a noticeable change in the number of TRPV1-expressing dorsal root ganglion cell bodies nor in the pattern of TRPV1 afferent terminal expression in the lumbar superficial dorsal horn between the mutant and wild-type mice. We appreciate, however, that we have not ruled out possible sprouting of individual TRPV1-positive afferents in the mutant mice. Sprouting could provide a more distributed heat input to the mispositioned NK1R neurons in Lamina I–II, the consequence of which would be greater heat pain information transfer to the brain and to spinal reflex withdrawal circuits. Taken together, we conclude that the increased heat pain sensitivity in the mutant mice reflects the increased number of NK1R-expressing neurons, many of which are mispositioned, but nevertheless receive a TRPV1 input comparable to that of the wild-type mice. Of course, an increase in the number of NK1R-expressing projection neurons would also enhance the feedforward spino-bulbo-spinal facilitatory loops that increase the excitability of nociresponsive neurons in deep dorsal horn (Rahman et al., 2008), many of which engage heat-induced withdrawal reflex circuitry. As both the hot plate and Hargreaves tests revealed increased sensitivity to heat in the mutant mice, we conclude that mispositioned NK1R-expressing projection and interneurons respond to the stimulation.

Unresolved is the question concerning the consequence of mispositioning of NK1R-expressing neurons of the LSN to Laminae I–II. Earlier studies reported that some LSN neurons, which unlike Lamina I neurons do not receive a direct input from primary sensory neurons, express NK1Rs, project to the ventrolateral medulla, and induce Fos expression in response to noxious thermal stimulation (Olave and Maxwell, 2004). Taken together with the present results we suggest that the heat hypersensitivity phenotype manifest in the mutant mice results from mispositioned NK1R neurons, some of which originated in the LSN and would therefore receive substance P inputs from dorsally located interneurons rather than primary sensory afferents (Gutierrez-Mecinas et al., 2018). Interestingly, Sikandar et al. (2017) found that LSN neurons predominantly process input from deep, rather than cutaneous tissues. In future studies, it will be of interest to assess the responsiveness of the mutant mice to deep tissue, including muscle, injury.

### Mispositioned NK1R-expressing neurons are functional

Importantly, our recording of heat-provoked Fos induction in mispositioned NK1R neurons in the superficial dorsal horn illustrates that the synaptic circuits targeted by the complement of TRPV1 afferents is maintained. This conclusion is consistent with other reports that mispositioned neurons in *Reln^-/-^* mice receive substantial functional synaptic input from the same sources as in wild-type mice (Caviness, 1976; Terashima et al., 1987; Yip et al., 2003), although the dendritic arbor of the mispositioned Dab1-labeled neurons is often stunted, as seen in the hippocampus (Niu et al., 2004). For example, retrograde and anterograde tracing studies comparing *Reln^+/+^* and the essentially inverted *Reln^-/-^* cerebral cortex found evidence of similar corticospinal (Terashima et al., 1983) and corticocortical (Caviness and Yorke, 1976; Terashima et al., 1985) connections, plus reciprocal connections of motor, somatosensory, and visual cortices with their appropriate thalamic targets (Caviness and Frost, 1983; Terashima et al., 1987). In the spinal cord, the supraspinal and primary afferent innervation of mispositioned *Reln^-/-^* sympathetic preganglionic neurons is also organized correctly (Yip et al., 2003). Thus, if migrating NK1R projection neurons from lateral Lamina V in a *Reln^-/-^*mouse failed to stop, and instead continued circumferentially to settle in Laminae I–II, these projection neurons would likely continue to receive substantial synaptic input from the rather large population of substance P-expressing interneurons (Liu et al., 1994; Xu et al., 2008; Dickie et al., 2019) .

### Specificity of dorsal horn circuits that process noxious heat and mechanical information

Of particular interest are the divergent effects of the *Reln*-signaling pathway mutations on heat and mechanical pain processing. Rats treated with SP-SAP and tested one month later had marked attenuation of both thermal and mechanical hyperalgesia in chronic pain models, but no changes detected in acute pain tests (Mantyh et al., 1997; Nichols et al., 1999). In contrast, we detected a small, but significant increase of heat pain thresholds after SSP-SAP treatment in the *dab1^+/+^* mice, possibly because SSP-SAP is a more effective toxin than SP-SAP (Wiley, 2008) or that mice differ in their response compared to rats (Mantyh et al., 1997; Nichols et al., 1999; Suzuki et al., 2002). Based on the previous SP-SAP studies, one would conclude that the NK1R cells contribute to and may be required for both heat and mechanical hyperalgesia. That may be the case in wild-type, but the results in *dab1^-/-^* mice demonstrate that activity of NK1R cells, even when their number is increased, does not lead to increased mechanical hypersensitivity. In fact, the *dab1^-/-^* mice have significantly decreased mechanical sensitivity.

In this regard, *Reln^-/-^* and *dab1^-/-^* mice bear considerable similarity to mice with a deletion of the testicular orphan nuclear receptor (TR4), which have increased mechanical thresholds and a profound loss of excitatory interneurons in the superficial dorsal horn, some of which express Reln (Wang et al., 2013). More recently, Szabo et al. (2015) reported decreased mechanical sensitivity in the conditional *Lmx1b* mutant mouse, which also is missing excitatory interneurons, including Reln-labeled cells. In addition, both of these mutants, as well as *Reln^-/-^* mice, have a reduction in the size of Laminae I–II outer and altered lamination patterns (Wang et al., 2013; Szabo et al., 2015; Yvone et al., 2017). Conceivably, there are comparable interneuronal abnormalities in *Reln^-/-^* mice that cause a loss of excitatory noxious mechanical input from mechanically responsive interneurons in the superficial dorsal horn. We previously demonstrated that ablation of the IB4 population of sensory neurons is associated with a remarkably selective increase in mechanical thresholds (Cavanaugh et al., 2009). Taken together, these results suggest that mispositioning of NK1R neurons does not underlie the increased mechanical thresholds in the *Reln^-/-^* mice. Rather, this deficit likely reflects circuit abnormalities among superficial dorsal horn interneurons that are relatively selective for the processing of mechanical pain information (Peirs et al., 2015; Petitjean et al., 2015; Peirs and Seal, 2016; Cheng et al., 2017). That conclusion is consistent with many recent studies emphasizing that, at least at the level of dorsal horn circuitry, there is considerable specificity in the processing of nociceptive heat, nociceptive mechanical and pruritoceptive information (Kardon et al., 2014; Petitjean et al., 2015; Cheng et al., 2017).

## Synthesis

Reviewing Editor: Esther Stoeckli, University of Zurich Decisions are customarily a result of the Reviewing Editor and the peer reviewers coming together and discussing their recommendations until a consensus is reached. When revisions are invited, a fact-based synthesis statement explaining their decision and outlining what is needed to prepare a revision will be listed below. Note: If this manuscript was transferred from JNeurosci and a decision was made to accept the manuscript without peer review, a brief statement to this effect will instead be what is listed below.

I have carefully looked at the revised manuscript and the responses you gave to the reviewers' comments. I do not agree that the manuscript does not contain any progress compared to previous publications. The finding that the behavior of the mice changes after ablation of NK1R+ is convincingly shown and adds new insight into our understanding of the mechanisms of pain and mechanical information processing in the spinal dorsal horn.

However, I feel that there are still some issues that you should address to make the paper stronger and suitable for publication.

Please confirm that the experiments have been carried out according to animal welfare regulations. What is the responsible agency that provided the permit and accepted the animal experimentation protocol.

What do the authors mean by “using pairs of [e.g.] dab+/+ and dab -/- mice”. I do not think that it is adequate to compare pairs rather than the results from the entire groups. Please comment on this rather strange wording.

The criticism of the statistics by reviewer 2 may be linked to the fact that it is not clear what has been compared. It would be easy to clarify by saying in the text and/or in the legend that the difference was x cells per section/hemisection taken from n mice. Maybe the numbers can sometimes be deduced from all the information given in axis label and legend but please make it easier on the reader.

Similarly, it would be helpful to have the graphical representation side-by-side rather than mixed in the same column. This would make it easier to see the differences in average value and the variability within the group.

Please indicate the size of the cohorts that were used for the behavioral tests.
